# Transorbital approach for a Meckel’s cave hybrid benign tumor: operative video and technical nuances

**DOI:** 10.3171/2025.1.FOCVID24164

**Published:** 2025-04-01

**Authors:** Alberto Di Somma, Alejandra Mosteiro, Lorena Gomez, Óscar Felguera, Joaquim Enseñat

**Affiliations:** Departments of 1 Neurological Surgery Hospital Clínic de Barcelona, Spain and; 2 Ophthalmology, Hospital Clínic de Barcelona, Spain

**Keywords:** endoscopic transorbital, transorbital surgery, skull base surgery, minimally invasive skull base

## Abstract

This video presents the transorbital endoscopic resection of a Meckel’s cave lesion, initially suspected as a trigeminal schwannoma. A 35-year-old woman with trigeminal hypoesthesia, pain, and gait instability underwent near-total tumor removal, with a transient cranial nerve III deficit that resolved completely. Histopathology identified the tumor as a hybrid benign lesion (trigeminal schwannoma/neurofibroma, WHO grade I). This minimally invasive technique offers effective tumor resection while preserving neurological function, making it a valuable option for select patients. Postoperative follow-up was recommended for ongoing assessment and care.

The video can be found here: https://stream.cadmore.media/r10.3171/2025.1.FOCVID24164

## Transcript

Hello everybody, in this video we will show a transorbital approach for a Meckel’s cave hybrid benign tumor, an operative video with technical nuances. Our patient is a 35-year-old female with hypoesthesia and pain along trigeminal nerve branches, especially V1 and V2, and gait instability. No relevant medical history is presented and no prior treatment. Neuroradiological investigations were performed via an MRI that you can see here. The lesion inside the Meckel’s cave is shown with a component in the middle fossa and another component in the posterior fossa. A cystic component displacing the brainstem toward the contralateral side.

The CT scan revealed partial erosion of the petrous apex, suggesting that an additional petrosectomy will likely not be required during surgery.

Different surgical strategies can be used with primary advantages and limitations. Retrosigmoid approach is a good choice to manage directly the posterior fossa lesion and the attachment to the brainstem, with the disadvantages to have the VII–VIII on the road. Middle fossa approach is a good point to get to the lesion, but some disadvantages related to the elevation and manipulation of the temporal lobe and the correlated veins. Another approach is the pretemporal surgical route that can be used to address from the anterior perspective this tumor, but may require some large skin incision and soft tissue manipulation. As well, a ventral endoscopic endonasal route can be used but, in this case, the internal carotid artery is displaced medially together with the cavernous sinus, so it should be crossed in order to get to the lesion. Another approach is the modified lateral orbitotomy approach that follows the long axis of the tumor without retracting the orbital content. So, in this context a transorbital endoscopic approach can be used in order to assess directly the anterior portion, the middle fossa portion of the lesion to get the posterior fossa following the long path of the lesion. So, taking into account advantages and limitations that are summarized in this table of each of the surgical routes, we decided for an endoscopic transorbital superior eyelid approach. So you can see here in the 3D reconstruction this is the route and the perspective of the approach. You can see the temporal muscle in pink, the optic nerve in orange, and the tumor at the end in yellow.

So, we chose for a transorbital approach due to its minimally invasive nature, direct access to Meckel’s cave, and ability to reduce brain retraction and manipulation. Additionally, the approach allows for a cosmetically favorable outcome with hidden incisions.

### 3:10 Skin Phase.

So, with the patient placed supine, a superior eyelid skin incision is made with monopolar Colorado, then the orbicularis oculi muscle is dissected and spared toward its fibers and then the periosteum covering the lateral orbital rim is cut in order to show the lateral orbital rim. Dissection proceeds inside the orbit and some stitches are placed in order to protect the skin. The lateral orbital rim is fully skeletonized and the cuts in the superior and inferior part are planned with the piezoelectric system, as you can see here.

### 3:47 Working Space Phase.

So, superior cut is performed, the inferior cut is performed, and then the inner cut with the high-speed drill is performed in order to detach the lateral orbital rim from the temporal muscle. Retractor is placed in order to move the orbit content toward the medial side.

Dissection and drilling proceed toward the depth of the surgical field in order to show the temporal dura. Lesser sphenoid wing and greater sphenoid wing are drilled out, and the sagittal crest is exposed and progressively removed, as you can see here. The sagittal crest represents the very medial end of the greater sphenoid wing. And then the horizontal part of the greater sphenoid wing representing the middle fossa floor is drilled down, as you can see here; the meningo-orbital band is coagulated and cut in order to permit elevation and manipulation of the temporal pole.

So, you can see now dissection of the temporal dura. Stitches are placed to elevate it and the tumor can be exposed, as you can see here.

### 4:53 Tumor Resection.

Now that the tumor is exposed it starts the tumor resection phase, as you can see coagulation of the tumor and resection with the CUSA ultrasound. And you can see also in this 3D reconstruction, the ventral perspective of the tumor presented in yellow here, and in blue the temporal lobe. Internal carotid artery (cavernous, lacerum, and petrous portion) are displaced medially and inferiorly. In the posterior part, you can see the brainstem. So dissection and removal proceed toward the posterior fossa. Now you can see we open up the cystic component, and at the end of tumor resection we found a very straight adhesion of the lesion in the pons, so we decided to leave it in place. As you can see here, the perspective of the surgical field at the end of tumor resection, and also in the 3D scan you can see the brainstem at the end of the surgical field. The temporal lobe and the lateral wall of the cavernous sinus and the middle fossa.

### 5:55 Reconstruction Phase.

So now starts the reconstruction phase that we used to do with autologous fat graft to cover the petrous apex in this case, and also to close the posterior fossa and trigeminal pore from the middle fossa, and fibrin glue and more fat graft inside the surgical cavity, as you can see here, to cover all the defect space. And also more fat graft is placed in the orbit cavity in order to prevent CSF leak and also enophthalmos. After that a stitch is placed in the superior part of the orbital rim to protect this part of the skin that is very very thin. While a screw and bar is placed in the inferior part of the lateral orbital rim, as you can see here. After that, together with our oculoplastic surgeon, skin incision is closed in multilayer fashion as you can see here. It is very important to close the periosteum of the lateral orbital rim and also to perform a Prolene continuous suture over the skin.

So now, let’s take a quick look at the anatomy of the region shown in the video from a transorbital perspective. Here, we can observe a right-sided transorbital approach with both interdural and extradural dissection of Meckel’s cave and the cavernous sinus. V1 and V2 are visible, with V3 seen more laterally. Using a dissector, we carefully open the Meckel’s cave, exposing the fibers of the Gasserian ganglion. Further dissection of the superficial dural layer of the Meckel’s cave allows us to follow the trigeminal pore posteriorly, providing access to the posterior fossa and a clear view of the brainstem. You can see here we are moving the endoscope from the middle fossa to the posterior fossa in order to enter the trigeminal pore and to see the brainstem.

Now, returning to the surgical case, postoperative MRI shows a near-total resection of the tumor, with a thin layer of the tumor capsule attached to the pons in the posterior fossa. And you can see also the surgical corridor filled with the autologous fat graft.

At the 6-month follow-up, the MRI shows an almost complete reabsorption of the autologous fat graft, with no complications observed and also a marked improvement in brainstem edema.

At late follow-up, the patient is in good condition, with no gait instability, preserved visual acuity in both eyes, a hidden skin incision at the level of the superior eyelid, and no trigeminal pain. However, persistent hypoesthesia along the trigeminal nerve branches is noted.

Histopathological examination identified the lesion as a hybrid benign tumor (trigeminal schwannoma/neurofibroma, WHO grade I) so postoperative follow-up was recommended.

Moreover, an evolutive CT scan demonstrates successful fusion of the superolateral orbital rim that was removed during surgery, as illustrated in this 3D reconstruction.

In conclusion, this case highlights the critical importance of tailoring surgical approaches to each individual patient. While various techniques are available, today the transorbital approach has emerged as a valuable option in modern surgical practice. Thank you.

**9:30 Related Papers on This Topic^1–10^**

## Disclosures

Dr. Di Somma reported being a consultant for Brainlab.

## Author Contributions

Primary surgeon: Di Somma, Felguera, Enseñat. Assistant surgeon: Di Somma, Mosteiro, Gomez, Felguera. Editing and drafting the video and abstract: Di Somma. Critically revising the work: Di Somma. Reviewed submitted version of the work: Di Somma, Mosteiro. Approved the final version of the work on behalf of all authors: Di Somma. Supervision: Di Somma, Enseñat.

## Supplemental Information

### Patient Informed Consent

The necessary patient informed consent was obtained in this study.
